# A Structure-free Method for Quantifying Conformational Flexibility in proteins

**DOI:** 10.1038/srep29040

**Published:** 2016-06-30

**Authors:** Virginia M. Burger, Daniel J. Arenas, Collin M. Stultz

**Affiliations:** 1 Research Laboratory for Electronics, Massachusetts Institute of Technology, 77 Massachusetts Ave. Cambridge MA 02139, USA; 2 Department of Physics, University of North Florida, 1 University of North Fl Dr, Jacksonville, FL 32224, USA; 3 Electrical Engineering and Computer Science & Institute for Medical Engineering and Science Massachusetts Institute of Technology, 77 Massachusetts Ave. Cambridge MA 02139, USA.

## Abstract

All proteins sample a range of conformations at physiologic temperatures and this inherent flexibility enables them to carry out their prescribed functions. A comprehensive understanding of protein function therefore entails a characterization of protein flexibility. Here we describe a novel approach for quantifying a protein’s flexibility in solution using small-angle X-ray scattering (SAXS) data. The method calculates an effective entropy that quantifies the diversity of radii of gyration that a protein can adopt in solution and does not require the explicit generation of structural ensembles to garner insights into protein flexibility. Application of this structure-free approach to over 200 experimental datasets demonstrates that the methodology can quantify a protein’s disorder as well as the effects of ligand binding on protein flexibility. Such quantitative descriptions of protein flexibility form the basis of a rigorous taxonomy for the description and classification of protein structure.

Thermally induced conformational fluctuations enable proteins to sample a range of structures under physiologic conditions. In many cases, this flexibility is required for a protein to carry out its prescribed function. Quantitative assessments of protein flexibility would therefore further our understanding of the relationship between protein function and structure.

The combination of experiment and computation forms a powerful platform for characterizing protein flexibility. Small-angle X-ray scattering (SAXS), in particular, is one popular experimental method that is often used in this context. Although SAXS typically yields low-resolution information, the combination of SAXS and atomistic simulations can provide insight into conformational changes in proteins and protein flexibility^1^,^2^,^3^,^4^. The ensemble optimization method (EOM) and the BILBOMD algorithm, for example, facilitate the construction of conformational ensembles for which the ensemble-averaged theoretical SAXS profile is in agreement with experimentally determined SAXS profiles^3^,^5^. The resulting conformational ensemble provides a rich dataset that can be used to study the role of protein flexibility in protein function.

Many existing approaches for gaining insight into structural flexibility from experimental data belong to a class of approaches that generate a set of structures to agree with a pre-specified set of experimental observations. This process of generating a set of protein structures that fit a given set of experimental measurements, however, is an underdetermined problem because the number of degrees of freedom in the protein is generally much larger than the number of experimental constraints. While this statement is applicable to all proteins, the problem is most egregious for disordered proteins that, by definition, sample a vast region of conformational space. For these systems there are often many different ensembles that agree with a given set of experimental observations^6^,^7^. These considerations raise the concern that conclusions arising from these methods may differ depending on the specific choice of the underlying structural model^6^. For example, structural ensembles generated with molecular dynamics simulations can differ depending on the choice of force field and/or solvent model, regardless of whether the protein of interest is disordered or not^8^,^9^. In addition, while modeling portions of the protein as rigid bodies serves as a useful method for reducing computational time (and is particularly useful for modeling multi-domain proteins)^10^,^11^,^12^, it is not always clear what regions of the molecule should, *a priori*, be constrained. The resulting ensemble will therefore depend on the manner in which one chooses to introduce constraints.

Consequently, there is a role for structure-free methods that provide information about protein flexibility. Quantitative metrics of protein flexibility calculated from the experimental data alone would facilitate objective comparisons between different proteins, while avoiding the introduction of biases due to the specific choice of structures or simulation protocol. Moreover, metrics that quantify protein flexibility provide a basis for a comprehensive classification scheme for protein structure^13^. Indeed, although proteins are typically categorized as being folded or unfolded, this distinction is overly simplistic because all proteins sample a range of structures at physiologic temperatures. Folded proteins have relatively homogenous ensembles, whereas unfolded proteins have relatively heterogeneous ensembles. Hence, quantitative metrics that provide insight into the heterogeneity within an underlying ensemble would provide a more complete view of the complexity that underlies protein structures and their thermal motions^13^.

In this work we describe a new formalism for quantifying protein flexibility from SAXS data. Our approach distinguishes between proteins that have different degrees of disorder and provides novel insights into ligand-induced effects on protein flexibility.

## Theory

### The Radius of Gyration Distribution (RgD) Model

The measured scattering intensity of a protein is the sum of the scattering intensities of all macromolecular conformations within the protein solution. Thus,


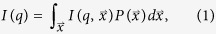


where *q* is the magnitude of the scattering vector, 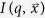 is the scattering intensity of the conformation denoted by 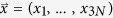, N is the number of atoms in the protein, and 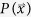 is the probability that the macromolecule has conformation 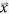. Protein flexibility/disorder can be quantified by calculating the entropy, which is a function of 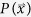. To compute the probability, 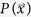, of any given conformation, the associated Boltzmann factor is required. Unfortunately, determining Boltzmann factors requires knowledge of the exact potential function and modern day empirical potential energy functions are not sufficient for estimating the true density of states under the precise experimental conditions of interest.

To simplify the calculation of the entropy, we propose a model that differentiates conformations based on their radius of gyration, instead of their conformation – a process that reduces the dimensionality of the problem from 3N degrees of freedom to one. Thus, we consider the probabilities of every possible radius of gyration, as opposed to every possible conformation, for our estimation of entropy. The radius of gyration criterion is convenient in SAXS experiments because in the low *q* region, where the intensity falls off by about one order of magnitude, the intensity is mainly dependent on the size of the macromolecule, i.e. its radius of gyration. We therefore propose a minimalist model in which the intensity profile of a conformation with radius of gyration *R*_*g*_ is represented by the intensity predicted for a sphere with homogeneous charge density^14^,^15^ – a quantity we denote by *I*_*S*_*(q, R*_*g*_) and derive in the Supplementary Information.

The scattering intensity in the context of the Radius-of-gyration Distribution (RgD) model, I_*μ,σ*_*(q)*, is given by


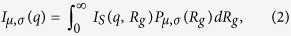


where *P*_*μ,σ*_(*R*_*g*_) is the probability distribution function (pdf) over the different radii of gyration that a protein can adopt in solution. The model uses a log-normal distribution for the pdf,


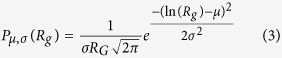


where μ and σ are the mean and standard deviation of the log-normal distribution. The log-normal distribution has the advantages that it is only defined for positive values of *R*_*g*_, and *P*(*R*_*g*_) approaches zero as *R*_*g*_ approaches zero. For practical use, we set *P*(0) = 0.

To fit the modeled scattering intensity, *I*_*μ,σ*_*(q)*, to the experimental scattering intensity, *I*_exp_*(q)*, we find values of μ and σ that minimize the difference between *I*_*μ,σ*_*(q)* and *I*_*exp*_*(q)*. More information on the minimization method is provided in the Supplementary Information. The optimal values of μ and σ are denoted as 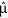 and 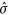. Using these values, the entropy S is computed as:


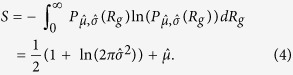


A consequence of equation [4] is that the entropy has a lower bound of −∞, a fact that distinguishes it from other discrete entropy measures (e.g., the Shannon entropy) for which the lower bound is zero. The difference in lower bounds between continuous and discrete probability distributions is emphasized by using the term “differential entropy” for the continuous case^16^. The differential entropy expressed by S is a quantitative estimate of the diversity of sampled radii of gyration in solution.

## Results and Discussion

### RgD on Model Systems

The RgD formalism uses a spherical model to calculate the scattering intensity of a given protein conformation. Modeling protein structures, and conformations within a disordered ensemble, by spheres is admittedly a simplification that does not capture the complexity inherent in the structures of biological molecules. However, we were encouraged by the fact that the use of simplified models of biological polymers has a long and rich history of providing important insights into many biological processes^17^,^18^,^19^,^20^,^21^,^22^,^23^,^24^,^25^. To determine whether the RgD formalism has the sensitivity needed to quantify protein flexibility using SAXS intensity profiles, we applied the method to model protein systems representing different degrees of disorder.

We began by choosing three proteins to study – one representing a folded, compact, protein, another a partially disordered protein, and the third an intrinsically disordered protein. Our overall approach was to construct ensembles for each protein, generate a theoretical ensemble average SAXS profile for each ensemble, and then input these data into our RgD algorithm to determine whether the RgD model can produce entropies that are consistent with our understanding of the relative disorder of these systems. In this sense, the constructed conformational ensembles are “reference ensembles”, from which experimental observables are calculated. For these simulated experiments the goal is not to generate ensembles that agree with some predefined set of experimental data. By contrast, the structural ensembles represent the “ground truth”, which is then used to calculate SAXS profiles. The resulting SAXS profiles are then input to the RgD algorithm to determine whether the method can differentiate proteins according to their flexibility.

For the folded protein we ran molecular dynamics simulations of the 202 residue bacterial toxin protein CcdB from the control of cell death and quiescence gene in *E. coli*^26^. For the partially unfolded protein, we chose the related bacterial antitoxin CcdA, a 144-residue dimer containing a folded core and two intrinsically disordered C-terminal tails, each 34 residues in length^27^. Lastly, for the disordered protein, we used a previously described ensemble for the 130-residue K18 fragment taken from the intrinsically disordered protein tau^6^. Ensemble average SAXS spectra for a given protein were calculated by first computing the individual SAXS spectrum for each structure using Crysol^28^ and then averaging the results. These proteins were chosen because they were the focus of prior studies in our group; i.e., structural libraries for these systems already existed. Details of the ensemble construction and calculation of the SAXS profile are discussed in the Supplementary Information.

**Figure 1:**
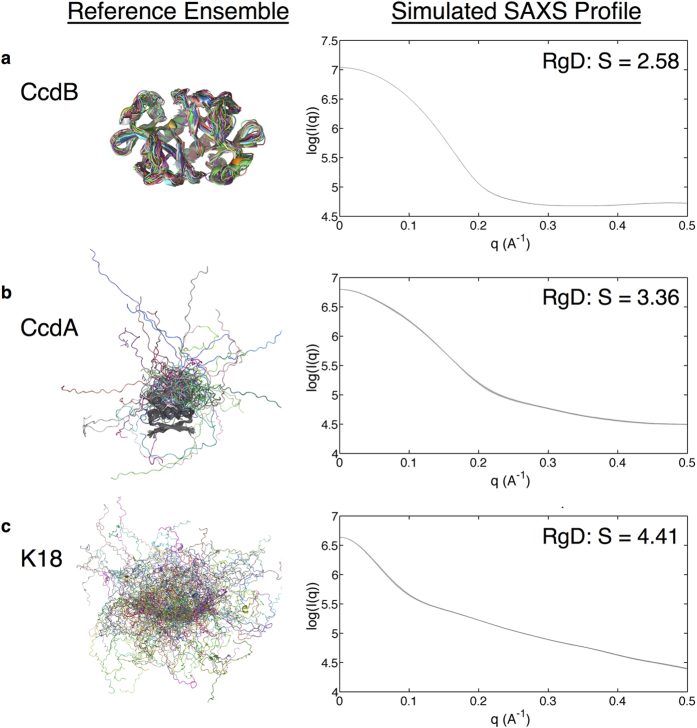
Results from calculations on simulated systems. Alignments of structures in each conformational ensemble are shown on the left. Simulated SAXS profiles and calculated RgD are also shown.

Results are shown in Fig. 1. The CcdB ensemble contains the least structural heterogeneity and has the lowest RgD entropy (S = 2.58, Fig. 1a). By contrast, the ensemble corresponding to the intrinsically disordered protein, K18, has the highest RgD entropy (S = 4.41, Fig. 1c), and the partially unfolded protein ensemble has an intermediate value (S = 3.36, Fig. 1b). To determine whether these RgD entropy values are significantly different, we estimated the error associated with RgD calculations using the reported errors in experimental scattering intensities (see Supplementary Information). In general, RgD entropy errors are less than 1% of the calculated entropy value. These data suggest that differences in the calculated RgD entropies shown in Fig. 1 cannot be attributed to experimental noise alone.

It is important to note that the RgD calculations do not utilize the structural ensembles themselves; i.e., RgD entropies are calculated from the SAXS spectra alone. To determine how our data compare to other structure based estimates that use both the structure and the SAXS profile, we used EOM to compute quantitative estimates of protein flexibility from each of the three model systems we considered^12^. As noted above, EOM takes a SAXS profile as input and generates a corresponding library of structures to arrive at a weighted ensemble of conformations that agree with the SAXS intensity profile. Once the ensemble is determined, the corresponding Shannon entropy provides a measure of the protein’s flexibility^12^. In prior applications, this quantity is referred to as *R*_*flex*_ and is typically represented as a percentage where 100% represents maximum flexibility^12^.

We used the EOM algorithm to generate a pool of 10,000 conformations for each system based on their amino acid sequences and then used these sequence-based conformational pools to fit the theoretical SAXS spectra using the genetic algorithm component of EOM^12^. The predicted *R*_*flex*_ values of the selected ensembles – 40% for CcdB, 55% for CcdA and 88% for K18 – showed a range of flexibilities in agreement with the values obtained with the RgD model; i.e., 2.58 for CcdB, 3.36 for CcdA and 4.41 for tau.

While structure-based metrics like *R*_*flex*_ are clearly useful for evaluating the flexibility of systems for which the conformational ensemble is unknown, they require the generation of a set of representative structures. Since RgD requires only a SAXS profile to produce an estimate of a system’s flexibility, it can provide additional information that may help guide the choice of structural library to use with structure based methods like EOM; e.g., proteins with large RgD entropies should have a large structural library that contains a wide range of different structures, while proteins with small RgD entropies may be better modeled as compact or folded.

### Kratky Plots and the RgD Entropy

Kratky plots of SAXS intensity data are commonly used for qualitative assessment of protein disorder. For compact proteins, *I(q)* will decay as *q*^*−4*^, whereas the scattering intensity of a flexible Gaussian chain will decay as *q*^*−2*^ or slower^29^. This suggests that the degree of protein disorder can be inferred from a visual inspection of a plot of *q*^*2*^*I(q)* versus *q*; i.e., a Kratky plot. Compact proteins will have *q*^*2*^*I(q)* values that approach zero (or baseline) at high *q*, while unfolded, or disordered, proteins will generally plateau at intermediate angles followed by continuously increasing values of *q*^*2*^*I(q)* at wide angles^1^,^30^,^31^.

**Figure 2:**
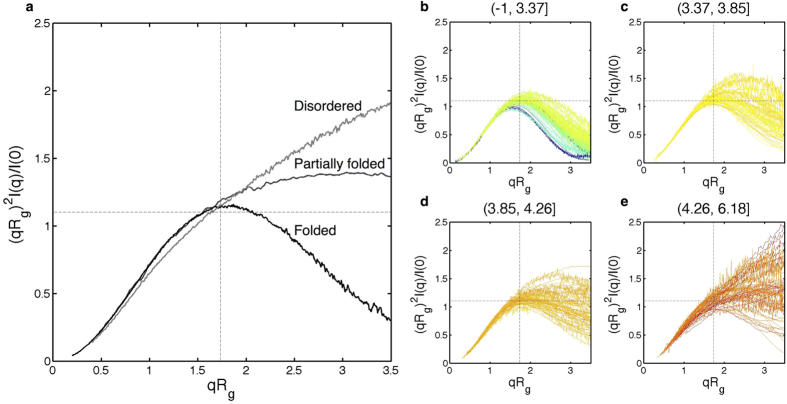
Dimensionless Kratky plots. Dotted lines are drawn at 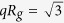 and *(qR*_*g*_)^*2*^*I(q)/I(0)* = 1.104. Folded proteins have a local maximum where the two lines intersect. (**a**) Disordered spectrum: C-terminal region of the Bromodomain adjacent to zinc finger protein domain 2B^62^; Partially folded spectrum: Splicing factor U2 Auxiliary Factor 65 KD (U2AF65), residues 148–475^52^; Folded spectrum: Chymotrypsinogen A^63^. (**b–e**) Dimensionless Kratky plots of 226 proteins from the BIOISIS^33^ and SASBDB^34^ databases organized into quartiles based on their entropy values. The entropy values are divided into four quartiles for the purpose of illustration. The plots are colored such that lower entropies are blue and higher entropies are red.

An alternate version of a Kratky analysis renders *(qR*_*g*_)^*2*^*I(q)/I(0)* versus *qR*_*g*_. The x- and y-axes of these plots are dimensionless and therefore are independent of the size and molecular weight of the molecule of interest. Hence these normalized or dimensionless Kratky plots are useful for the analysis of SAXS profiles across different systems. An additional advantage of this formalism is that the dimensionless Kratky plot of a well-folded biopolymer will have a local maximum at 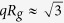, which is given by *(qR*_*g*_)^*2*^*I(q)/I(0)* = *3e*^*−1*^ = 1.104. Homogeneous solutions of folded polymers therefore have dimensionless Kratky plots that have an identifiable characteristic shape^32^. Deviations from this ideal behavior suggest that the macromolecule has conformational flexibility. In Fig. 2a, characteristic dimensionless Kratky plots for spectra from disordered, partially folded and folded proteins are shown.

To assess how results obtained with the RgD model compare to a Kratky analysis, we calculated entropy values for biopolymers in the BIOISIS database^33^ and the Small Angle Scattering Biological Database (SASBDB)^34^. Available entries from either database were excluded from our analysis if: 1) the sample used to obtain the SAXS profile was reported to be aggregated or unpurified; 2) the entry corresponds to unpublished data; or 3) the scattering profile only sampled *q* values less than 0.3 Å^−1^. This latter requirement ensured that each entry had enough data to perform a meaningful analysis using standard approaches such as a Kratky plot. This screen left a total of 226 experimental datasets for our analysis (Supplementary Tables S1 and S2).

Figure 2b–e show normalized Kratky plots for the datasets in our analysis, divided into four quartiles according to the entropy (S) computed by RgD. The entropy values are divided into four quartiles for the purpose of illustration only. Entropy values vary between −1 and 6.18, where entries that fall in the lowest quartile (S ≤ 3.37) have dimensionless Kratky plots that are characteristic of compact, folded, states (Fig. 2b). By contrast, dimensionless Kratky plots in the highest quartile (S > 4.26) are characteristic of flexible or disordered biopolymers (Fig. 2e). Entropy values between 3.37 and 4.26 correspond to intermediate behavior, with values between 3.86 and 4.27 associated with relatively increased flexibility (Fig. 2c,d).

It is important to recognize that the RgD model was not designed to simply quantify the information contained in Kratky plots. Indeed, since Kratky plots can be difficult to interpret and are sometimes unable to provide an accurate assessment of protein flexibility^35^,^36^, a simple reproduction of insights obtained from a Kratky analysis should not, in and of itself, be the sole metric of success^37^. To demonstrate that the model provides information that is distinct, and complementary, to existing SAXS based methods for the assessment of protein flexibility, we used the model to quantify ligand-induced changes in protein flexibility.

### The RgD Entropy and Ligand-Binding

We began by searching the BIOISIS database to find a suitable subset of protein-ligand complexes for additional analyses^38^. Only entries where both the spectra of the free and complexed protein were obtained by the same research group, and under similar experimental conditions, were considered. Below we discuss our results below, in light of the available experimental data.

#### MnmE

*E. coli* MnmE plays a crucial role in modifying wobble uridine in tRNA^39^. In separate studies, X-ray crystallography, electron paramagnetic resonance (EPR), and SAXS experiments were used to study the structure of MnmE in 1) the free state, 2) bound to the transition state analogue GDP-A1Fx, and 3) bound to the ground state analogue GppNHp^40^,^41^. In the free state MnmE adopts an open structure where two of its domains (the G-domains) are separated, while binding to GDP-A1Fx causes the protein to adopt a “closed” conformation where the G-domains dimerize^41^. By contrast, binding to GppNHp induces the protein to adopt a mixture of closed and open conformations, where approximately 88% of the protein is in the closed state and 12% is in the open state^40^.

**Figure 3:**
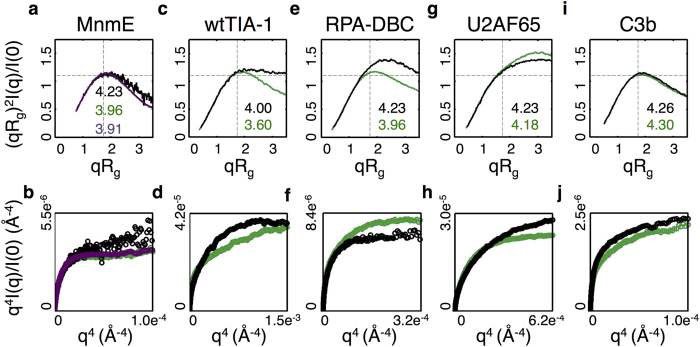
Dimensionless Kratky plots (top row), calculated RgD entropy values (insets in top row), and Porod Debye plots (bottom row) for MnmE: E. coli MnmE in isolation (black) and bound to GppNHp (green), and GDP-AlFx (purple); wtTIA-1: The alterative splicing factor wtTIA-1 RRM123 in the absence of RNA (black) and bound to 11-nucleotide AU-rich segment taken from the 3′-untranslated region of tnf-α (green); RPA-DBC: The DNA-binding core of heterotrimeric Replication protein A in the absence (black) and presence (green) of a 30 nucleotide ssDNA substrate; U2AF65: U2 auxiliary factor residues 148-475, in the absence (black) and presence of RNA (green); C3b: Complement fragment C3b in the unbound (black) state and bound to the extracellular fibrinogen binding protein (Efb) from S. aureus (green). Since we work with normalized Intensity profiles (that are divided by *I(0)*) the y-axis of each Porod-Debye plots is divided by *I(0)*.

Dimensionless Kratky representations of the three systems are very similar in that all three proteins have a local maximum at 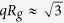, and at this value *(qR*_*g*_)^*2*^*I(q)/I(0)* = *3e*^*−1*^ = 1.104 (Fig. 3a). It is therefore difficult to make any conclusions about the relative stability of these complexes from these data alone. Given that the dimensionless Kratky plots provide little, if any, insight into ligand-induced changes in protein flexibility, we performed a Porod-Debye analysis to determine how this approach compares to the RgD model. The Porod-Debye relationship dictates that for a compact polymer the scattering intensity decays as *q*^*−4*^ and that for some small range of *q*, a plot of *q*^*4*^*I(q)* vs. *q*^*4*^ will achieve a plateau, which is a function of the molecule’s surface area and its electron density contrast with respect to the surrounding solvent^37^,^42^,^43^. In practice, the range of *q* where the Porod-Debye law is applicable – the Porod-Debye region – is estimated from the position of the first peak in the corresponding Porod plot (i.e., *q*^*4*^*I(q)* vs *q*). Proteins that have considerable flexibility decay slower than *q*^−*4*^ and therefore do not reach a plateau in the Porod-Debye region.

A Porod-Debye analysis does clarify the role of flexibility to some degree. The unbound protein does not have a clear Porod-Debye plateau (Fig. 3b, black), while the bound proteins do (Fig. 3b, green and purple). These data suggest that binding of both GDP-A1Fx and GppNHp reduces MnmE flexibility. However, it is not clear from these data which analog causes the greatest reduction in flexibility after binding. Without additional information it is difficult to make conclusive statements about relative protein flexibility from these observations.

The RgD model suggests that binding of both the ground state analog and the transition state analog reduces the flexibility of the protein and that binding of the transition state analog, GDP-A1Fx, is associated with the greatest reduction in flexibility (Fig. 3a). Moreover, as errors in the experimental scattering intensities correspond to small errors in the calculated RgD entropy values (approximately 0.03 for free MnmE and 0.01 for MmmE bournd to GppNHp, and 0.003 for MnmE bound to GDP-A1Fx, see Supplementary Table S3), it is difficult to ascribe the differences in RgD values between the three systems to experimental error alone.

Since GDP-A1Fx binding causes the protein to adopt a closed state, these observations argue that the closed state is the most rigid. The fact that the MnmE-GppNHp complex has an intermediate value for the entropy is consistent with the observation that GppNHp binding leads to an equilibrium distribution of closed and open states^40^,^41^.

#### wtTIA-1 RRM123

T-cell intracellular antigen-1 (wtTIA-1) plays a crucial role in pre-mRNA splicing and is an important regulator of translation^44^. It contains three RNA recognition motifs (RRMs) that bind U-rich RNA segments downstream of other weak splice sites. Recently the binding of all three RRMs (wtTIA-1 RRM123) to U-rich RNA sequences was studied using SAXS and isothermal titration calorimetry (ITC)^45^,^46^.

Dimensionless Kratky plots of wtTIA-1 RRM123 in its free and bound state suggest that binding is associated with a loss of protein flexibility. The Kratky plot for the bound state (Fig. 3c, green) has a local maximum, which is close to the ideal value for a folded polymer, relative to the plot corresponding to the unbound state (Fig. 3c, black). However, Porod-Debye plots of wtTIA-1 RRM123 yield contradictory information (Fig. 2d, black). While the free protein reaches a clear Porod-Debye plateau by *q*^4^ ≈ 0.18^4^ = 0.001 Å^4^, the plateau is lost in the bound state (Fig. 2d, green). A plot of *q*^*3*^*I(q)*^*3*^ vs. *q*^*3*^ for the bound state further demonstrates that *I(q)* decays as *q*^*−3*^ in the Porod-Debye region instead of the expected *q*^−*4*^ for a compact polymer, thereby suggesting that binding makes the protein more flexible (Supplementary Fig. S1)^37^.

ITC studies suggest that RNA binding to wtTIA-1 is associated with large unfavorable changes in the binding entropy (approximately 30 kcal/mol)^46^. In general, the total binding entropy is a function of several different physical phenomena including, for example, dynamical changes in the binding species, release of ordered water molecules, and the vibrational spectra of both the bound and unbound states^47^. The RgD model suggests that RNA binding is associated with a decrease in the entropy (Fig. 3c), and therefore argues that a decrease in conformational entropy contributes to the large unfavorable entropic contribution to the binding energy. While there is certainly precedent for ligand binding to increase the conformational entropy of a protein^48^,^49^, as the Porod-Debye plots suggest, the large unfavorable entropy associated with RNA binding is more consistent with a loss of protein flexibility^46^,^50^, as the RgD model suggests.

#### RPA-DBC

Replication protein A (RPA) is multi-domain protein that plays an important role in regulating DNA processing. Recently a combination of SAXS and molecular dynamics simulations was used to study binding of the DNA-binding core of RPA (RPA-DBC) to a 30-nucleotide ssDNA substrate^51^. Extensive simulations were performed to generate structures that were consistent with experimentally determined SAXS profiles of the free and bound protein. A conformational analysis of the resulting ensembles suggested that RPA-DBC bound to ssDNA is more compact relative to the free protein and that the bound state samples a smaller range of radii of gyration relative to the unbound protein. These observations are echoed by our calculations in that binding to DNA leads to a decrease in the RgD entropy (Fig. 3e). Since the RgD model quantifies the diversity of sampled radii of gyration, a decrease in the RgD entropy means that the bound state samples a smaller range of radii of gyration in solution.

The dimensionless Kratky plots are also consistent with these data in that the plot of the bound protein has a peak located at the ideal position for a folded protein, whereas the free protein does not (Fig. 3e). A Porod-Debye plot of the bound complex has a clear plateau (Fig. 3f, green), and at first glance a similar plot for the free protein plateaus as well, albeit to a lower value (Fig. 3f, black). The fact that both plots plateau to different values suggests that the free and bound structures have different spectroscopic properties. Since flexibility cannot be inferred from the value of the plateau itself, it is unclear how these observations relate to any changes in protein flexibility^37^. It could be argued that the Porod-Debye plot of the free protein slowly increases at relatively wide angles (*q*^4^ > 0.00025 ≈ 0.125^4^ Å^4^, Fig. 3f, black), but this may be secondary to experimental noise (or poor buffer subtraction) – phenomena that may be seen at higher *q* values^31^. Indeed, at high-*q* the scattering profile of the free protein has larger variations than that of the bound complex (Supplementary Fig. S2). In short, it is difficult to reconcile observations arising from this Porod-Debye analysis with the results of the combined SAXS/simulation study mentioned above. In this regard, the RgD model provides clarifying information that complements the results of the Kratky and Porod-Debye analyses.

#### U2AF65

The splicing factor U2AF65 assembles on RNA during the early stages of pre-mRNA splicing. During assembly U2AF65 binds to pre-mRNA at the 3′ splice site. Recently the binding of the SF1/U2AF65 Splicing Factor Complex was studied using SAXS^52^. Experiments with U2A65 utilized a construct (residues 148–475) containing three domains: one that recognizes the N-terminal region of splicing factor 1; and two RNA recognition domains, each of which bind RNA^53^. Dimensionless Kratky plots of U2AF65 suggest that both the unbound and bound states are flexible (Fig. 3g). Given that the individual domains are known to be folded, these data are consistent with U2AF65 being composed of folded modular domains that are connected by flexible linkers^52^. Nevertheless, it is difficult to make definitive statements about the relative flexibility of the bound state from these data alone. The RgD model predicts that binding leads to a decrease in the system entropy (Fig. 3g). However, it should be mentioned that the decrease is small and very close to the errors in entropy that we estimated using noise simulations (see Supplementary Table S1). A Porod-Debye plot of the bound state of the U2AF65 spectrum has a plateau (Fig. 3h, green) relative to its free state (Fig. 3h, black), suggesting that binding results in a decrease in the system entropy, a finding consistent with the RgD results.

#### C3b

The complement fragment C3b plays an important role in human immunity^54^. Interactions of C3b trigger a host of inflammatory responses that eventually lead to the death of foreign microorganisms. Binding of C3b to the extracellular fibrinogen-binding (Efb) protein from *S. aureus* was recently studied using a combination of SAXS and molecular modeling^55^. Dynamical simulations of C3b were conducted to generate a minimal set of conformers that agreed with SAXS profiles of the protein in its free and bound forms. The resulting ensembles suggest that C3b samples both open and closed states in its unbound form. In the open state, two domains of C3b (the CUB and TED domains), which are connected to the core of the protein via a flexible linker, adopt conformations that are separated from the core. By contrast, in the closed state, the CUB-TED domains are packed against the protein core. A combination of hydrogen-deuterium exchange experiments and molecular simulations suggest that Efb binds at the interface between the TED domain and protein core, and that Efb binding stabilizes the protein in the open state^55^.

Dimensionless Kratky plots of the free and bound protein are very similar (Fig. 3i) and the associated Porod-Debye plots do not plateau, making it unclear whether binding has any influence on flexibility (Fig. 3j). The RgD entropy calculations suggest that both the free and bound proteins are very flexible in that their RgD entropy values place them in the third and fourth quartiles of proteins in the BIOISIS and SASBDB databases (Figs 3i and 2d,e). Moreover, the calculated entropy for the bound state is larger than the entropy of the free protein, suggesting that the bound protein is more flexible than the unbound protein. However, it should be noted that the difference between these values are quite small and within the range of error associated with RgD calculations (n.b. the errors associated with RgD calculations on C3b are 0.01, as shown in Supplementary Table S3). Since binding of Efb stabilizes the open state, these calculations suggest that the bound, and predominantly open, state is able to sample a range of radii of gyration that is similar to, or possibly larger than, that of the unbound protein.

The aforementioned simulations argue that the free protein samples closed and open states that have similar radii of gyration and that the measured radius of gyration of the free protein is a weighted sum over these values^55^. Similarly, the RgD entropy, which is calculated from the RgD model, is also a weighted sum of entropic contributions from both the closed and open states. If the open state were more flexible than the closed state, then stabilization of the open state through binding by Efb would result in an increase in the overall entropy. The dynamical simulations mentioned above utilized a protocol where the CUB-TED domains were modeled as rigid bodies connected by flexible linkers, with the rest of the protein held in a fixed position. In light of this, it is difficult to gauge the relative flexibilities of the open and closed states, and how binding affects the flexibility of the open state, from these calculations. Nonetheless, the entropy computed for the bound and unbound SAXS profiles with RgD allows us to predict that Efb binding to the open states results in the protein sampling a wider range of radii of gyration.

## Conclusions

A number of experimentally derived metrics have been developed to quantify protein flexibility. For example, quantitative metrics that facilitate the study of protein flexibility include X-ray diffraction at different temperatures^56^, NMR relaxation experiments^57^,^58^, and atomic force microscopy^59^. These approaches, however, often require experimental conditions that are quite different from the solution state, or the use of isotopically labeled protein. In addition, these experiments only account for motions that occur on the microsecond-to-millisecond time scales. SAXS, albeit a low-resolution technique, has the advantage that it provides information about the structure of the protein in solution without the use of special isotopes, and also provides information about large conformational changes that typically occur on long time scales^3^,^60^.

Our approach estimates the pdf over the different radii of gyration that a biomolecule can adopt in solution using the SAXS profile alone. Once the pdf is known, the entropy can be calculated in a straightforward manner. Since the entropy computed by RgD quantifies the diversity of radii of gyration sampled by a protein in solution, this method provides a direct measure of a system’s disorder. Application to over 200 proteins in the BIOISIS^33^ and SASBDB^34^ databases demonstrates that the RgD model can provide information about the degree of a protein’s disorder, as well as insight into how ligand binding affects protein flexibility.

The RgD entropy is a continuous parameter that quantifies the extent of disorder in a protein’s conformational ensemble; i.e., the set of thermally accessible conformations available in solution. It is our view that such quantitative descriptions of protein structure are more accurate than the traditional binary terms, “folded” and “unfolded”, which are often used to classify proteins. Indeed, a more accurate description of protein structure should entail a characterization of the heterogeneity within a protein’s conformational ensemble^13^. The importance of this realization is highlighted by the fact that not all folded proteins are created equal. Some “folded” ensembles are more heterogeneous than others, as evidenced by the range of RgD entropies that are observed for different folded proteins (Fig. 2b). Similarly, disordered proteins often exhibit preferences for particular structural features^61^. These considerations reinforce the notion that quantitative metrics describing the heterogeneity within a protein’s ensemble provide a more comprehensive assessment of protein structure than binary classification.

## Additional Information

**How to cite this article**: Burger, V. M. *et al.* A Structure-free Method for Quantifying Conformational Flexibility in proteins. *Sci. Rep.* **6**, 29040; doi: 10.1038/srep29040 (2016).

## Supplementary Material

Supplementary Information
