# Supplementary material for: A Structure-free Method for Quantifying Conformational Flexibility in proteins
Source: Sci Rep. 2016 Jun 30;6:29040. doi: 10.1038/srep29040 (PMC4928179; doi:10.1038/srep29040)
Supplement: Supplementary Information [file srep29040-s1.pdf]

A Structure-free Method for Quantifying Conformational Flexibility in proteins

Virginia M. Burger[1*], Daniel J. Arenas[1*], & Collin M. Stultz[1,2]

Supplementary Information

1. The Scattering Intensity of a Sphere with Homogeneous Charge Density, $I_S\left(q,R_g\right)$

The scattering intensity of a sphere with uniform charge density can be derived using the Fourier-transform, in spherical coordinates, of a homogeneous sphere with charge density ρ_0 and radius R_S:

$$I_S\left(\vec{q},R_S\right)=\left|\int_{r=0}^{R_S}\int_{\theta=0}^{2\pi}\int_{\varphi=0}^{\pi}\rho_0 e^{-i\vec{q}\cdot\vec{r}}\,dV\right|^2. \qquad [1]$$

Here the scattering vector, $\vec{q}$, corresponds to the change in momentum between the incoming and outgoing wave-vectors, $\vec{r}=(r,\theta,\varphi)$ denotes a point in the sphere, and dV is the volume element. Without loss of generality, we can choose the direction of $\vec{q}$ to coincide with the z-axis. With this convention we have $\vec{q}\cdot\vec{r}=|\vec{q}||\vec{r}|\cos\varphi$. Using the fact that $dV=r^2\sin\varphi\,d\varphi d\theta dr$, we obtain:

$$I_S\left(q,R_S\right)=\left|\int_{r=0}^{R_S}\int_{\theta=0}^{2\pi}\int_{\varphi=0}^{\pi}\rho_0 e^{-iqr\cos\varphi}r^2\sin\varphi\,d\varphi\,d\theta\,dr\right|^2 \qquad [2]$$

where $q=\left|\vec{q}\right|$. Note that the right hand side of equation [2] makes it clear that the intensity of a spherically symmetric object, with a homogeneous charge density, is only a function of the magnitude of the scattering vector. Integration over the azimuthal angle, θ, yields a factor of 2π and integration over the polar angle, φ, yields:

$$\int_{\varphi=0}^{\pi} e^{-iqr\cos(\varphi)} \sin(\varphi)\, d\varphi = \frac{2\sin(qr)}{qr}. \qquad [3]$$

The intensity therefore reduces to:

$$\begin{aligned} I(q,R_S) &= (4\pi\rho_0)^2 \left| \int_{r=0}^{R_S} \frac{\sin(qr)}{qr} r^2\, dr \right|^2 \\ &= \frac{(4\pi\rho_0)^2}{q^6} \left| \int_{u=0}^{qR_S} u \sin(u)\, du \right|^2 \quad \text{where } u = qr \\ &= \left(\frac{4\pi\rho_0}{q^3} \right)^2 \left[\sin(qR_S) - qR_S \cos(qR_S) \right]^2. \end{aligned} \qquad [4]$$

To obtain the final result we need to obtain an expression for the intensity at q=0. At low values of q, we perform a Taylor series expansion, keeping only the first order terms of the right hand side of equation [4] to obtain:

$$\begin{aligned} I_S(q \to 0) &\approx \left(\frac{4\pi\rho_0}{q^3} \right)^2 \left[\left(qR_S - \frac{q^3 R_S^3}{3!} \right) - qR_S \left(1 - \frac{q^2 R_S^2}{2!} \right) \right]^2 \\ &= \left(\frac{4\pi\rho_0}{q^3} \right)^2 \left[\frac{q^3 R_S^3}{3} \right]^2 \\ &= \left(\frac{4\pi R_S^3 \rho_0}{3} \right)^2 \end{aligned} \qquad [5]$$

Hence as the q approaches zero, the intensity approaches the square of the total charge of the sphere. Therefore we rewrite equation [4] as follows:

$$I_S(q) = I_S(0) \frac{9}{(qR_S)^6} \left[\sin(qR_S) - qR_S \cos(qR_S) \right]^2 . \qquad [6]$$

Using the fact that $R_G^2 = \frac{3}{5} R_S^2$, we obtain:

$$I_S\left(q,R_g\right)=I_S(0)\frac{9}{\left(q\alpha R_g\right)^6}\left[\sin\left(q\alpha R_g\right)-q\alpha R_G\cos\left(q\alpha R_g\right)\right]^2, \qquad [7]$$

where $\alpha=\sqrt{\frac{5}{3}}$ and $I_S(0)$ is the intensity at $q=0$.

2. Finding optimal values for $I_S(0)$, μ and σ

Solving the R_gD model entails finding optimal parameters for μ, σ and $I_S(0)$ that minimize $\varepsilon\left(\mu,\sigma,I_S(0)\right)$:

$$\varepsilon\left(\mu,\sigma,I_S(0)\right)=\int_0^\infty\frac{\left(I_{\exp}(q)-I_{\mu,\sigma}(q)\right)^2}{\varepsilon_{\exp}(q)^2}, \qquad [8]$$

where $I_{exp}(q)$ is the experimentally determined scattering intensity, $\varepsilon_{\exp}(q)$ is the associated error, and $I_S(0)$ appears in the expression for $I_{\mu,\sigma}(q)$ (see equation [7]). Furthermore, since experimental error estimates are not available for all BIOISIS entries, we set $\varepsilon_{\exp}(q)=1$ in equation [8] to ensure that all of the scattering profiles would be treated equally.

A natural choice for $I_S(0)$ is the experimental scattering intensity at $q=0$. However, in SAXS measurements one cannot measure directly at $q=0$ due, in part, to the difficulty that arises from the non-zero width of the incoming beam. We therefore use Guinier's law to exploit the analytical behavior at small q, thereby obtaining an estimate for the experimental scattering intensity at $q=0$. More precisely, at small q the scattering intensity can be approximated as follows[1]:

$$I(q)=I(0)e^{-\frac{R_g^2q^2}{3}}. \qquad [9]$$

This approximation is the basis of the widely used Guinier plots, where the natural logarithm of $I(q)$ is plotted versus q^2, and a line is fit to the data at low values of q. The slope of the line is used to compute the average radius of gyration, which serves as our initial guess for μ in our optimization algorithm, and the y-intercept is used as the estimate of $I(0)$. Once the value of $I(0)$ is known, we normalize the entire intensity spectrum by this value, i.e. $I_{Normalized}(q) = I(q)/I(0)$. Normalization facilitates the comparison of the different intensity profiles that are obtained using different protein concentrations and different experimental setups. This is particularly important because the intensity at $q = 0$ is, in general, a function of the protein concentration, the X-ray source intensity, and the sensitivity of the detector. After normalization we set $I_S(0) = 1$.

Once initial values for $I_S(0)$ and μ are chosen, we perform a grid-search minimization where we vary σ values from 0.01 to 1 in increments of 0.01. The value of σ that minimizes $\varepsilon(\mu,\sigma)$, where μ is the value obtained from Guinier's law, is used as the initial value in a gradient-descent algorithm.

In the first step of the gradient-descent algorithm we fix $I_S(0) = 1.0$ and then search for values of μ and σ that minimize $\varepsilon(\mu,\sigma,I_S(0))$. We then perform another set of minimizations where all three variables are allowed to vary to find the minimum value for $\varepsilon(\mu,\sigma,I_S(0))$. This stepwise procedure generally leads to faster convergence rates.

3. Guinier analyses

As outlined above, solving the RgD model involves first performing a Guinier analysis on the

scattering data. An important part of the Guinier analysis is to choose a region to linearly fit the data. Unfortunately the choice of this region can be problematic. Although a plot of the natural logarithm of $I(q)$ versus q^2 will be linear in the limit where q is smaller than the inverse dimensions of the protein, this condition does not translate to a well-defined cutoff in all cases. In our program, RgD, the user manually chooses the upper cutoff for the q^2 axis of the Guinier plot; then, a linear fit is performed in the range from the lowest q data available up to the specified cutoff.

The RgD program also has an automated option that iteratively calculates the cutoff. First, an initial value for the radius of gyration is calculated as a function of the number of amino acids N. For this function, a phenomenological constant capturing the relationship between R_g and N was determined by plotting R_g against $N^{1/3}$ for all entries in the BIOISIS database. Using the initial estimate, the first cutoff in the Guinier Plot is calculated as $(1.3/R_g)^2$; then R_g is calculated again using the new cutoff, and the process is continued until the calculations converge within a tolerance of 10^{-4}. In the study of complexes, both manual and automated options for the cutoff were used, and the calculated entropies differed by less than 1%.

4. Estimating the error in RgD calculations of entropy

It is also important to investigate the effect of experimental noise on the calculated entropy. This is particularly important for comparison of two systems measured in similar experimental conditions. Because we do not have the raw data for the entries in the databases, we simulate raw data for each entry by using the reported standard deviation of the experimental scattered intensity provided in the database entries; we back-simulate the raw data. To do this, at each q in the spectrum, the noise was calculated by using a random Gaussian distribution that had a

standard deviation equal to that of the experimental scattered intensity (at that particular q). Five noise-simulated spectra were generated for each system and the statistics of the entropy were calculated. Table S3 shows the results for representative datasets from the Bioisis and SASBDB databases.

5. RgD on Model Systems

We demonstrate the RgD model on three model systems using theoretical SAXS profiles. We chose the intrinsically disordered K18 tau isoform (130 residues), the partially disordered protein CcdA (144 residues, with two 34 residue intrinsically disordered regions), and the folded protein CcdB (202 residues) (Fig. 3). The Bayesian Weighting algorithm was used to generate an ensemble for the K18 tau isoform (available through http://www.rle.mit.edu/cbg/data.htm)[2]. The 77 structures from this ensemble which had a 5% probability of having a weight of at least 0.005 were used for this analysis. NMR models for the CcdA protein in two states were used as an ensemble for CcdA (PDB entries 2adl (1 model, open state), 2adn (1 model, closed state), 2h3a (20 models, open state), and 2h3c (20 models, closed state))[3]. A total of 1.6 microseconds of all-atom explicit solvent molecular dynamics simulations were generated for CcdB, initiated using conformations of CcdB extracted from crystal structures of CcdB bound to two distinct partners (PDB entries 1x75 and 3g7z)[4,5]. After 100ns of equilibration time for each structure, conformations were selected every 5ns along the trajectories to generate an ensemble of conformations sampled by CcdB. Details of the molecular dynamics simulations on CcdB are discussed in the next section.

For each ensemble, theoretical SAXS profiles were generated in Crysol, primarily using the default parameters [6]. The order for the Fibonacci grid was set to its maximal value to improve the

surface representation. The computed theoretical curves had 100 points ranging from q=0 $Å^{-1}$ to q = 0. 5 $Å^{-1}$. [6]. The ensemble-averaged mean SAXS profile was then computed, together with the standard errors over the ensemble. For K18 tau, the Boltzmann weights provided by the Bayesian weighting algorithms were used to compute the ensemble average. For CcdA and CcdB, the conformations were assumed to be equally probable.

The RgD algorithm was run for each theoretical SAXS profile with both a user-determined maximum q^2 and an automatically-determined maximum q^2. The intensity computed for q=0 $Å^{-1}$ was deleted prior to running RgD. The user-determined maximum q^2 was found in Primus by searching for the largest q such that $qR_g < 1.3$ [7]. The automatically determined maximum q^2 values were within 0.0007 of the manually determined values, and the resulting RgD entropy values were identical.

The ensemble-optimization method, implemented as EOM, was used to compute R_{FLEX} [8]. The genetic algorithm component of EOM (GAJOE, Genetic Algorithm Judging Optimisation of Ensembles) was run using a set of 10,000 random conformations that were generated based on the primary sequence of each protein by EOM. For these calculations, all default parameters were selected, with the exception that a compact-chain was selected for CcdB, the folded protein in our data set, and native-like chains were selected for the remaining proteins.

6. MD simulations of CcdB

The modeler software and the SCWRL4.0 side-chain prediction algorithm were used to model missing residues and atoms in CcdB structures, and chain termini were ionized [9-11]. For each of the two simulations, the CcdB structure was solvated in a dodecahedral box of TIP3P water, so

that the minimum distance between the protein and the box wall was 1nm. Sodium and Chloride ions were added so that the concentration was 150mM with neutral charge. MD simulations were performed in the NVT ensemble using the charmm27 force field in Gromacs 5.0.4[12-16]. Particle-mesh Ewald (PME) and periodic boundary conditions were used to compute long-range electrostatic interactions[17]. The temperature was stabilized at 300K using the V-rescale thermostat, a modified version of the Berendsen coupling thermostat, with separate coupling for the protein and the solvent[18]. The LINCS algorithm was used to constrain the lengths of all bonds, allowing a simulation time-step of 2fs[19]**.** Before solvating, each structure was minimized in vacuum using steepest descent minimization until the maximal force was less than 500kJ/(mol nm) or a maximum of 500 steps. A second round of steepest descent minimization was performed after minimization until the maximal force was less than 100 kJ/(mol nm) or a maximum of 50,000 steps. A 100ps molecular dynamics simulation was then performed for which the protein was position-restrained, to allow time for the solvent to equilibrate. The position-restraints on the protein were then removed, and a 100ns simulation was performed to equilibrate the system. Finally, a production simulation of approximately 700ns was performed for each CcdB structure. During the equilibration and production simulations, one atom was fixed so that the protein did not diffuse outside of its box. Structures were extracted every five nanoseconds from the trajectories to generate an ensemble of 285 conformations accessible to CcdB.

Table S1: BIOISIS entries used in our analysis

Entry	Description
http://www.bioisis.net/experiments/1	Ferredoxin NADP reductase from Pyrococcus furiosus
http://www.bioisis.net/experiments/2	Folded Dimer of PF0699
http://www.bioisis.net/experiments/3	Fusion Protein of Folded PF1282 and unfolded PF1205
http://www.bioisis.net/experiments/4	Annotated NADH oxidase, ORF PF0715
http://www.bioisis.net/experiments/5	Folded Trimeric PF1787.
http://www.bioisis.net/experiments/6	Monomeric PF1674.
http://www.bioisis.net/experiments/7	TetramericPF1281 – Superoxide Reductase
http://www.bioisis.net/experiments/8	Pyruvate: ferredoxin oxidoreductase (POR)
http://www.bioisis.net/experiments/9	Yeast tRNA-Phe (no proteins in sample)
http://www.bioisis.net/experiments/11	tRNA-Like sequence from Brome Mosaic Virus (no proteins in sample)
http://www.bioisis.net/experiments/12	Native 30S ribosomal subunit from S. solfataricus
http://www.bioisis.net/experiments/16	Native Yeast tRNA-Phe from Sigma (no proteins in sample)
http://www.bioisis.net/experiments/17	PF1282 Rubredoxin from P. furiosus.
http://www.bioisis.net/experiments/18	Bound Lysine Riboswitch from T. maritime (no proteins in sample)
http://www.bioisis.net/experiments/19	Lysine Riboswitch (no proteins in sample)
http://www.bioisis.net/experiments/20	Unbound lysine riboswitch from T. maritime (no proteins in sample)
http://www.bioisis.net/experiments/27	PF1950 Pyrococcus furiousus.
http://www.bioisis.net/experiments/28	PF2047.1Pyroccocus furiosus ORF 2047.1
http://www.bioisis.net/experiments/29	PF0230 Mixed Multimeric State of recombinant Pyrococcus furiousus ORF2030.
http://www.bioisis.net/experiments/30	PF1372 Pyrococcus furiousus.
http://www.bioisis.net/experiments/31	PF1061 Pyroccocus furiosus monomeric product.
http://www.bioisis.net/experiments/32	PF1033 Pyrococcus furiosus decameric product
http://www.bioisis.net/experiments/33	PF0706.1 Uncharacterized protein from Pyrococcus furiosus
http://www.bioisis.net/experiments/34	PF1291. Uncharacterized protein from Pyrococcus furiosus
http://www.bioisis.net/experiments/35	PF0021 Pyrococcus furiousus monomeric product
http://www.bioisis.net/experiments/36	PF0863. Pyrococcus furiousus dimeric product
http://www.bioisis.net/experiments/38	PF1528 - Pyrococcus furiosus monomeric product
http://www.bioisis.net/experiments/39	PF0553 – Pyrococcus furiousus monomeric product
http://www.bioisis.net/experiments/40	P4-P6 domain at 7.6 mM MgCl2 (no proteins in sample)
http://www.bioisis.net/experiments/42	SAM-1 Riboswitch (apo-state) (no proteins in sample)
http://www.bioisis.net/experiments/44	Abscisic Acid Binding to Dimeric PYR1
http://www.bioisis.net/experiments/45	Splicing factor SF1, residues 1-255
http://www.bioisis.net/experiments/46	Splicing factor SF1, residues 1-255 bound to RNA
http://www.bioisis.net/experiments/47	Splicing factor U2AF65, residues 148-375
http://www.bioisis.net/experiments/49	Splicing factor U2AF65, residues 148-375, bound to RNA.
http://www.bioisis.net/experiments/51	DNA-pKcs. Annotated: 40 bp stem loop DNA
http://www.bioisis.net/experiments/52	Ectodomain of the human SidT1 glycoprotein
http://www.bioisis.net/experiments/53	Ketoreductase-enoylreductase didomain

http://www.bioisis.net/experiments/54	Splicing factor complex of (P)SF1 residues 1-255, U2AF65 148-375 and ADMLRNA.
http://www.bioisis.net/experiments/55	wtTIA-RRM123, residues 1-247<br>Data collected at ALS
http://www.bioisis.net/experiments/56	wtTIA-RRM123, residues 1-247 bound to RNA<br>Data collected at ALS
http://www.bioisis.net/experiments/58	28bp DNA duplex (no proteins in sample)
http://www.bioisis.net/experiments/59	Domains 1 and 2 of tyrosine phosphatase LAR3.
http://www.bioisis.net/experiments/60	Glucose Isomerase under differing ionic strengths using NaCl
http://www.bioisis.net/experiments/61	Glucose Isomerase under differing ionic strengths using KCl
http://www.bioisis.net/experiments/62	Lysozyme Standard Curve.
http://www.bioisis.net/experiments/63	Lysozyme under differing ionic strengths using NaCl
http://www.bioisis.net/experiments/64	Lysozyme under differing ionic strengths using KCl
http://www.bioisis.net/experiments/65	Complement fragment C3b with extracellular fibrinogen binding protein.
http://www.bioisis.net/experiments/66	Glucose isomerase
http://www.bioisis.net/experiments/67	DNA double-strand break repair protein MRE11 + ATP
http://www.bioisis.net/experiments/68	Ubiquitin-like modifier-activating enzyme ATG7 C-terminal domain.
http://www.bioisis.net/experiments/69	Superoxide dismutase from Alvinella Pompejana
http://www.bioisis.net/experiments/70	Glycosyl hydrolase + C-terminus
http://www.bioisis.net/experiments/72	Splicing Factor complex PSF1. Residues 1-255 and U2AF65 residues 148-375
http://www.bioisis.net/experiments/73	Human regulator of Chromosome Condensation (RCC1)
http://www.bioisis.net/experiments/74	Annotated: Rab1 adenlyation (AMPylation) protein
http://www.bioisis.net/experiments/75	Crystal structure of bifunctional proline untilizaaion A flavoenzyme....
http://www.bioisis.net/experiments/76	Cu,Zn superoxide Dismutase from the pathogen Neisseria meningitidis
http://www.bioisis.net/experiments/77	Cu,Zn superoxide Dismutase from the pathogen Brucella abortus
http://www.bioisis.net/experiments/78	Median and Mean Xylanase Data
http://www.bioisis.net/experiments/80	RPA DNA-Binding Core
http://www.bioisis.net/experiments/81	RPA DNA-Binding Core with 30mer
http://www.bioisis.net/experiments/83	Complement fragment C3b
http://www.bioisis.net/experiments/84	Interleukin (IL)-33 with primary receptor ST2.
http://www.bioisis.net/experiments/85	MnmE in the nucleotide free state
http://www.bioisis.net/experiments/86	U2AF6 RM1-RRM2 with N- and C- terminal flanking sequences
http://www.bioisis.net/experiments/88	A. aeolicus MnmG + tRNA.
http://www.bioisis.net/experiments/89	Non-toxic hemaglotunnin. PH = 8.0
http://www.bioisis.net/experiments/90	U2AF6 RM1-RRM2.
http://www.bioisis.net/experiments/91	Botulinum Neurotoxin A at pH = 8.0

http://www.bioisis.net/experiments/92	Non-toxic non-hemagglutinin A at pH = 6.0
http://www.bioisis.net/experiments/93	Botulinum Neurotoxin A at pH = 6.0.
http://www.bioisis.net/experiments/94	Botulinum Neurotoxin A minimally functional progenitor toxin complexes (M-PTC) at pH=6.0
http://www.bioisis.net/experiments/98	BTB-CUL3 ubiquitin ligase with 30 residue N-terminal extension
http://www.bioisis.net/experiments/103	Complement fragment C3b with extracellular fibrinogen binding protein from S. aureus (Structural Model built using ENSEMBLE algorithm)
http://www.bioisis.net/experiments/104	Allosteric inhibition of C3b complement function by Efb staphylococal immune evasion protein
http://www.bioisis.net/experiments/105	E.coli MnmG + Nb_MnmG_1
http://www.bioisis.net/experiments/108	MnmE bound to GppNHp
http://www.bioisis.net/experiments/110	MnmE bound to GDP-AIFx
http://www.bioisis.net/experiments/113	PER2 PAS-AB Dimer.

Table S2: SASBDB entries used in our analysis

Entry	Description
http://www.sasbdb.org/data/SASDA32/	BSA
http://www.sasbdb.org/data/SASDA52/	Alcohol dehydrogenase
http://www.sasbdb.org/data/SASDA62/	Beta-Amylase
http://www.sasbdb.org/data/SASDA72/	beta Glucosidase
http://www.sasbdb.org/data/SASDA82/	Apoferritin in PBS and glycerol
http://www.sasbdb.org/data/SASDA92/	Catalase
http://www.sasbdb.org/data/SASDAA2/	Conalbumin
http://www.sasbdb.org/data/SASDAB2/	Cyt-C
http://www.sasbdb.org/data/SASDAC2/	Lyz in Sodium Acetate
http://www.sasbdb.org/data/SASDAG2/	Lysozyme in Sodium Acetate
http://www.sasbdb.org/data/SASDAH2/<br>http://www.sasbdb.org/data/SASDAK2/	Myoglobin<br>(Concentrations in the range 0.80-25.00 mg/ml were measured)
http://www.sasbdb.org/data/SASDAL2/	Ovalbumin
http://www.sasbdb.org/data/SASDAQ2/	Ubiquitin-60S ribosomal protein L40 in Na Acetate
http://www.sasbdb.org/data/SASDAN2/<br>http://www.sasbdb.org/data/SASDAR2/	RNase in PBS<br>(concentrations in the range 6.40-22.80 mg/ml)
http://www.sasbdb.org/data/SASDAX2/	Pyruvate decarboxylase
http://www.sasbdb.org/data/SASDAN3/	DNA mismatch repair protein MutS_dimer
http://www.sasbdb.org/data/SASDAR3/	Functional binding region (187-385) of the pneumococcal serine-rich repeat protein
http://www.sasbdb.org/data/SASDAS3/	Immunoglobulin- like filamin two-domain fragment 16-17
http://www.sasbdb.org/data/SASDAT3/	Immunoglobulin- like filamin two-domain fragment 18-19
http://www.sasbdb.org/data/SASDAU3/	Immunoglobulin- like filamin two-domain fragment 22-23
http://www.sasbdb.org/data/SASDAV3/	Geminin:Cdt1 2:1 heterotrimer
http://www.sasbdb.org/data/SASDAW3/	Geminin:Cdt1 4:2 heterohexamer
http://www.sasbdb.org/data/SASDAQ3/<br>http://www.sasbdb.org/data/SASDAX3/<br>http://www.sasbdb.org/data/SASDAY3/<br>http://www.sasbdb.org/data/SASDAZ3/<br>http://www.sasbdb.org/data/SASDA24/	DNA mismatch repair protein_MutS tetramer (data obtained at different protein concentrations)
http://www.sasbdb.org/data/SASDA54/<br>http://www.sasbdb.org/data/SASDA74/<br>http://www.sasbdb.org/data/SASDA84/	RNA Aptamer (data obtained from several protein concentrations)
http://www.sasbdb.org/data/SASDA94/	Der p21
http://www.sasbdb.org/data/SASDAA4/	Full length GbpA
http://www.sasbdb.org/data/SASDAB4/	Truncated GbpA
http://www.sasbdb.org/data/SASDAC4/	Filamin C 23-24
http://www.sasbdb.org/data/SASDAD4/	Full length GtBP3
http://www.sasbdb.org/data/SASDAE4/	C-terminal CtBP3

http://www.sasbdb.org/data/SASDAF4/	Drosophila melanogaster peroxisomal multifunctional enzyme type 2
http://www.sasbdb.org/data/SASDAG4/	Human peroxisomal multifunctional enzyme type 2
http://www.sasbdb.org/data/SASDAH4/	DH-PH module of PDZRhoGEF
http://www.sasbdb.org/data/SASDAJ4/	Exportin-1
http://www.sasbdb.org/data/SASDAK4/	Exportin-1 + GTP-binding nuclear protein Ran
http://www.sasbdb.org/data/SASDAL4/	Exportin-1 + GTP-binding nuclear protein Ran + Snurportin-1
http://www.sasbdb.org/data/SASDAM4/	Exportin-1 + Snurportin-1
http://www.sasbdb.org/data/SASDAN4/	Calmodulin + C-terminal region of human myelin basic protein
http://www.sasbdb.org/data/SASDAP4/	Chitinase 60
http://www.sasbdb.org/data/SASDAQ4/	Calmodulin + Glutamate decarboxylase
http://www.sasbdb.org/data/SASDAR4/	Polypyrimidine tract-binding protein 1
http://www.sasbdb.org/data/SASDAS4/	I27-PimA Fusion protein
http://www.sasbdb.org/data/SASDAT4/	Urokinase plasminogen activator surface receptor
http://www.sasbdb.org/data/SASDAU4/	Urokinase plasminogen activator surface receptor H47C/N259C
http://www.sasbdb.org/data/SASDAV4/	Urokinase plasminogen activator surface receptor (uPAR) + synthetic peptide AE105
http://www.sasbdb.org/data/SASDAW4/	uPAR + synthetic peptide AE234
http://www.sasbdb.org/data/SASDAX4/	uPAR + ATF Complex
http://www.sasbdb.org/data/SASDAY4/	Factor H CCP modules 10 to 15
http://www.sasbdb.org/data/SASDAZ4/	Factor H CCP modules 11 to 14
http://www.sasbdb.org/data/SASDA25/	Factor H CCP modules 12 to 13
http://www.sasbdb.org/data/SASDA35/	High-affinity zinc transporter periplasmic component Zinc/cadmium-binding protein
http://www.sasbdb.org/data/SASDA45/	Psi-producing oxygenase A
http://www.sasbdb.org/data/SASDA55/	Nucleoplasmin
http://www.sasbdb.org/data/SASDA65/	Nucleoplasmin-H5 complex
http://www.sasbdb.org/data/SASDA75/	Nucleoplasmin-H2AH2B complex
http://www.sasbdb.org/data/SASDA85/	Human Chromatin Remodeler CHD4 (363-1353)
http://www.sasbdb.org/data/SASDA95/	Human Chromatin Remodeler CHD4 (494-1353)
http://www.sasbdb.org/data/SASDAA5/	Human Chromatin Remodeler CHD4 (685-1233)
http://www.sasbdb.org/data/SASDAB5/	Human Chromatin Remodeler CHD4 (363-682)
http://www.sasbdb.org/data/SASDAC5/	Importin alpha/beta
http://www.sasbdb.org/data/SASDAD5/	Nucleoplasmin + Importin alpha/beta
http://www.sasbdb.org/data/SASDAE5/	CYNEX4 FRET probe, (eYFP-AnnexinA4-eCFP)
http://www.sasbdb.org/data/SASDAF5/	CYNEX4 FRET probe, (eYFP-AnnexinA4-eCFP) T266D mutant
http://www.sasbdb.org/data/SASDAG5/	RNA chaperone Hfq
http://www.sasbdb.org/data/SASDAH5/	RNA chaperone Hfq + RNA DsrA
http://www.sasbdb.org/data/SASDAJ5/	Annexin-A4
http://www.sasbdb.org/data/SASDAK5/	Myomesin-1
http://www.sasbdb.org/data/SASDAL5/	Clostridium difficile bacteriophage 27 endolysin

http://www.sasbdb.org/data/SASDAM5/	Clostridium difficile bacteriophage 27 endolysin C238R mutant
http://www.sasbdb.org/data/SASDAN5/	Cytochrome C + Adrenodoxin
http://www.sasbdb.org/data/SASDAP5/	Cytochrome C dimer + Adrenodoxin dimer
http://www.sasbdb.org/data/SASDAQ5/	Lumazine Synthase
http://www.sasbdb.org/data/SASDAR5/	ProNGF
http://www.sasbdb.org/data/SASDAS5/	aD11 Fab
http://www.sasbdb.org/data/SASDAT5/	NGF
http://www.sasbdb.org/data/SASDAU5/	aD11 Fab + NGF
http://www.sasbdb.org/data/SASDAV5/	apo XMRV RT
http://www.sasbdb.org/data/SASDAW5/	apo XMRV RT + RNA_DNA hybrid substrate
http://www.sasbdb.org/data/SASDAX5/	Endophilin-A1 BAR domain + arachidonyl-CoA
http://www.sasbdb.org/data/SASDAY5/	Endophilin-A1 BAR domain
http://www.sasbdb.org/data/SASDAZ5/	Netrin-1
http://www.sasbdb.org/data/SASDA26/	DCC56
http://www.sasbdb.org/data/SASDA46/	Bromodomain adjacent to zinc finger domain protein 2A
http://www.sasbdb.org/data/SASDA56/	Bromodomain adjacent to zinc finger domain protein 2B, C-terminal
http://www.sasbdb.org/data/SASDA66/	Bromodomain adjacent to zinc finger domain protein 2B, C-terminal + H3Kac9Kac14
http://www.sasbdb.org/data/SASDA76/	Netrin-1 + DCC56 (FN5 & FN6)
http://www.sasbdb.org/data/SASDA86/	Netrin-1 + Deleted in Colorectal Cancer (FN5 & FN6) M933R mutant
http://www.sasbdb.org/data/SASDA96/	Lysozyme C
http://www.sasbdb.org/data/SASDAA6/	Human serum albumin monomer and mixtures
http://www.sasbdb.org/data/SASDAB6/<br>http://www.sasbdb.org/data/SASDAK6/	Xylose Isomerase
http://www.sasbdb.org/data/SASDAF6/	K1K2K3 adhesin modules of lysine-specific (Kgp) gingipain
http://www.sasbdb.org/data/SASDAG6/	K1K2 adhesin modules of lysine-specific (Kgp) gingipain
http://www.sasbdb.org/data/SASDAH6/	bifunctional kinase- methyltransferase WbdD(1-459)
http://www.sasbdb.org/data/SASDAJ6/	bifunctional kinase- methyltransferase WbdD(1-556)
http://www.sasbdb.org/data/SASDAR6/	Macrophage colony-stimulating factor 1 +<br>Macrophage colony-stimulating factor 1 receptor
http://www.sasbdb.org/data/SASDAS6/	Plectin, fragment of the plakin domain encompassing the spectrin repeats SR3-SR4-SR5 and the SH3
http://www.sasbdb.org/data/SASDAT6/	Integrin beta-4
http://www.sasbdb.org/data/SASDAV6/	Cysteine desulfurase IscS_
http://www.sasbdb.org/data/SASDAW6/	Iron-sulfur cluster assembly scaffold protein IscU
http://www.sasbdb.org/data/SASDAX6/	Protein CyaY
http://www.sasbdb.org/data/SASDAY6/	Cysteine desulfurase IscS_<br>Iron-sulfur cluster assembly scaffold protein IscU
http://www.sasbdb.org/data/SASDAZ6/	Cysteine desulfurase IscS_

	Protein CyaY
http://www.sasbdb.org/data/SASDA27/	Cysteine desulfurase IscS<br>Iron-sulfur cluster assembly scaffold protein IscU<br>Protein CyaY
http://www.sasbdb.org/data/SASDA37/<br>http://www.sasbdb.org/data/SASDA47/<br>http://www.sasbdb.org/data/SASDA57/<br>http://www.sasbdb.org/data/SASDA67/<br>http://www.sasbdb.org/data/SASDA77/<br>http://www.sasbdb.org/data/SASDA87/	Surface Protein G (SasG) EG5 repeat protein G51-G52 (different protein concentrations used)
http://www.sasbdb.org/data/SASDA97/	PlaB
http://www.sasbdb.org/data/SASDAA7/	Histidine protein kinase_Response regulator_
http://www.sasbdb.org/data/SASDAB7/	Complex ComE-comcde_Response regulator_
http://www.sasbdb.org/data/SASDAC7/	Complex LytTR-comcde
http://www.sasbdb.org/data/SASDAG7/	Hyaluronate binding domain of CD44 antigen<br>Single-chain Variable Fragment of Antibody MEM-85
http://www.sasbdb.org/data/SASDAS7/	mouse olfactomedin-1
http://www.sasbdb.org/data/SASDA28/	anti-TG2 antibody (679 14 E06)
http://www.sasbdb.org/data/SASDA38/	transglutaminase-2 (TGA2)
http://www.sasbdb.org/data/SASDA48/	transglutaminase2:anti-transglutaminase2 FAB1 antibody complex
http://www.sasbdb.org/data/SASDA58/	UL26N of pseudorabies virus
http://www.sasbdb.org/data/SASDA68/	Fructose-bisphosphate aldolase A
http://www.sasbdb.org/data/SASDA78/	Carbonic Anhydrase
http://www.sasbdb.org/data/SASDA88/	RAID3
http://www.sasbdb.org/data/SASDA98/	Thyroglobulin
http://www.sasbdb.org/data/SASDAA8/	Chymotrypsinogen A
http://www.sasbdb.org/data/SASDAB8/	PRKCA-binding protein
http://www.sasbdb.org/data/SASDAC8/	SDS hydrolase SdsA1
http://www.sasbdb.org/data/SASDAD8/	Antiapoptotic membrane protein, (DpV84gp022) Deerpox virus
http://www.sasbdb.org/data/SASDAC9/	Varkud Satellite (VS) ribozyme
http://www.sasbdb.org/data/SASDB52/	Death associated protein kinase (wild-type)
http://www.sasbdb.org/data/SASDB62/	Death associated protein kinase (D220K mutant)
http://www.sasbdb.org/data/SASDB72/	Death associated protein kinase (Basic Loop mutant)
http://www.sasbdb.org/data/SASDBT2/	Ankyrin repeat domains from human Tankyrase-2 (489-649)
http://www.sasbdb.org/data/SASDBU2/	Human Arpin (isoform 1)
http://www.sasbdb.org/data/SASDBV2/	Zebrafish Arpin
http://www.sasbdb.org/data/SASDBW2/	Zebrafish (Danio rerio) Arpin truncated C-terminal mutant (delta-C 16)
http://www.sasbdb.org/data/SASDBX2/	Zebrafish Arpin in complex with the ankyrin repeat domains of human Tankyrase 2 (489-469)

Table S3: Estimate of the effect of noise on the uncertainty of the RgD entropy. The noise simulations were performed by using the reported standard deviation for the scattered intensity at each q. *The noise simulations for C3b were performed using the standard deviations of the experimental scattered intensity provided for C3b/Efb.

System	Calculated entropies after noise-simulation
MnmE	S = 4.22 +/- 0.03
MnmE/GppNHP	S = 3.96 +/- 0.01
MnmE/GDP-A1Fx	S = 3.91 +/- 0.003
wtTIA-1	S = 4.01 +/- 0.03
wtTIA-1/RNA	S = 3.60 +/- 0.01
RPA-DBC	S = 4.23 +/- 0.01
RPA-DBC/DNA	S = 3.96 +/- 0.01
U2AF65	S = 4.23 +/- 0.02
U2AF65/RNA	S = 4.18 +/- 0.03
C3b	S = 4.26 +/- 0.01*
C3b/Efb	S = 4.30 +/- 0.01

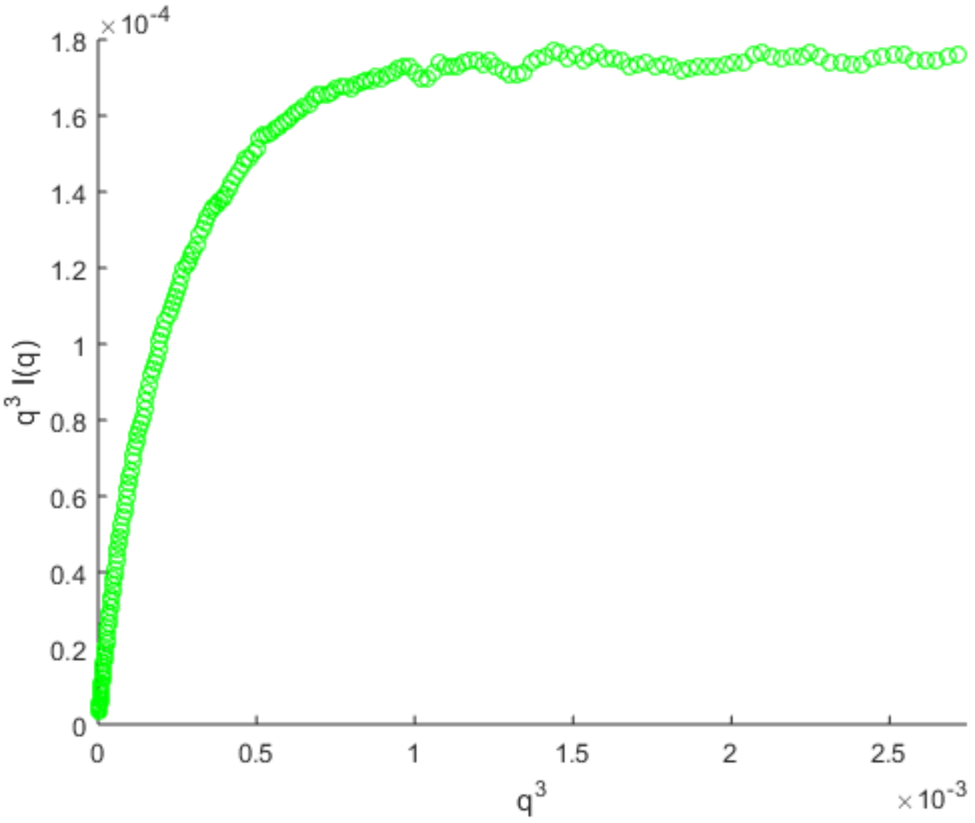

Figure S1: $q^3I(q)$ vs q^3 for wtTIA-RRM123, residues 1-247 bound to RNA. Units are Angstrom^3.

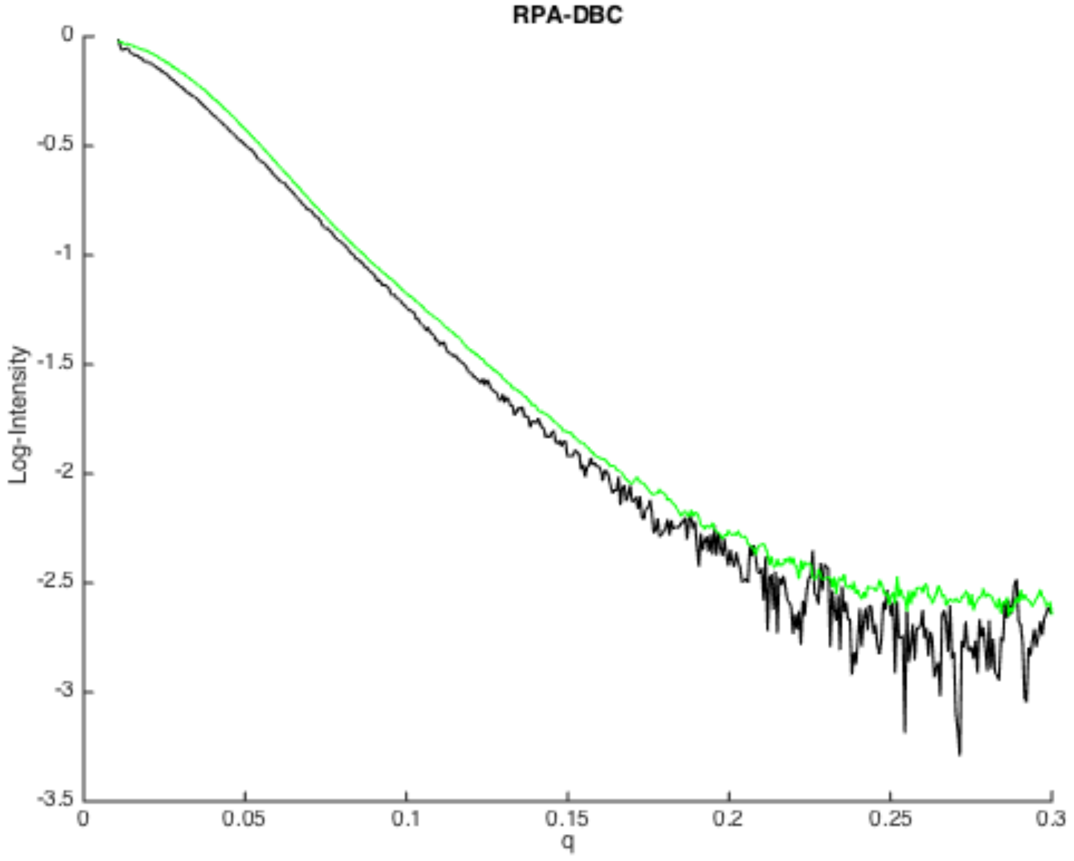

Figure S2: Scaled scattering profiles for RPA-DBC in its free (black) and bound (green) states. X-axis units are units are Angstrom.

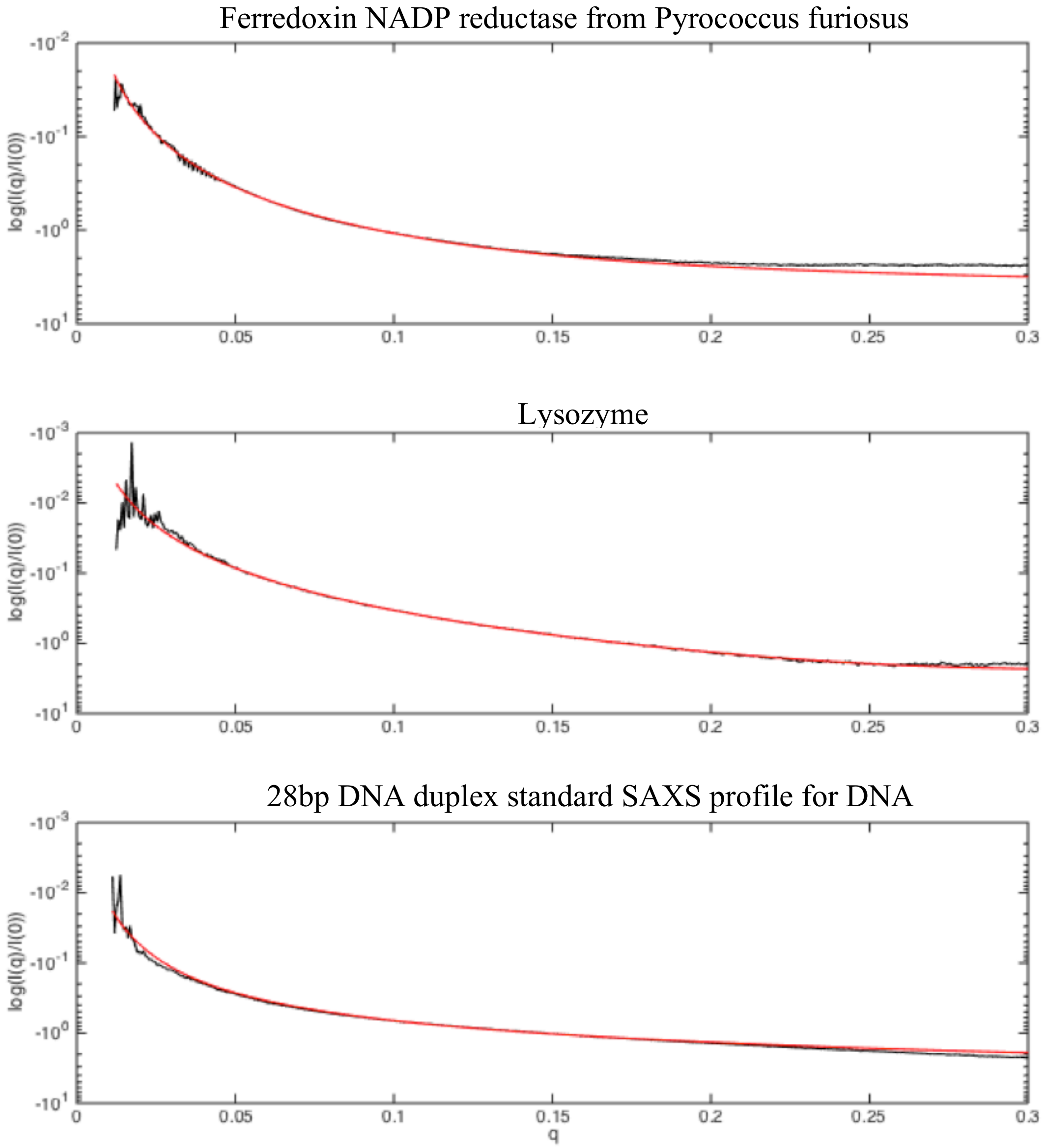

Figure S3: Representative RgD fits (red) to experimental data taken from BIOISIS (black). X-axis units are units are Angstrom.

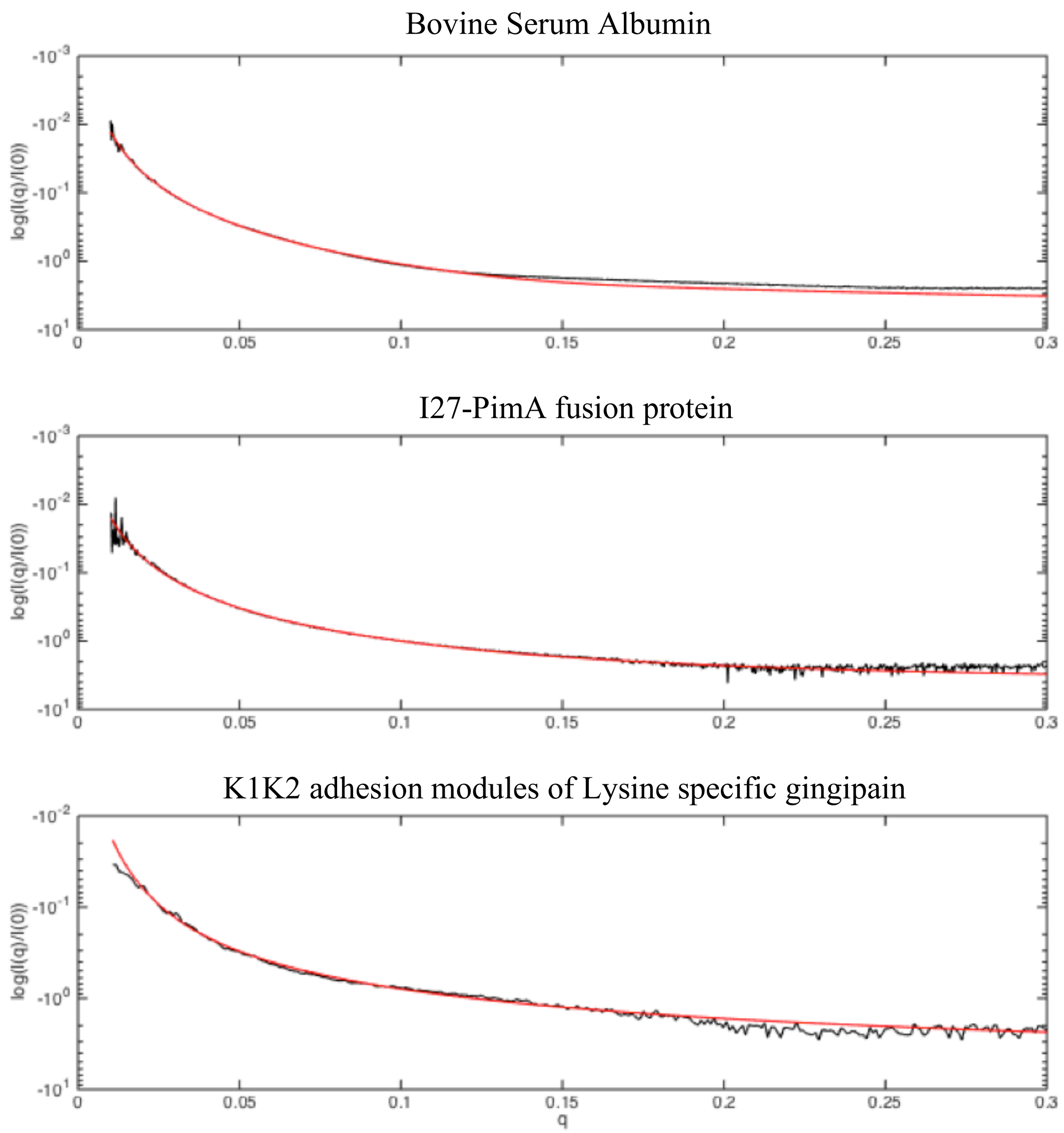

Figure S4: Representative RgD fits (red) to experimental data taken from SASBDB (black). X-axis units are units are Angstrom.

References

1 Guinier, A. & Fournet, G. Correction of measurements of low-angle X-ray scattering. *Nature* **160**, 501 (1947).

2 Fisher, C. K., Huang, A. & Stultz, C. M. Modeling Intrinsically Disordered Proteins with Bayesian Statistics. *J Am Chem Soc* **132**, 14919-14927, doi:10.1021/ja105832g (2010).

3 Madl, T. *et al.* Structural basis for nucleic acid and toxin recognition of the bacterial antitoxin CcdA. *J Mol Biol* **364**, 170-185, doi:10.1016/j.jmb.2006.08.082 (2006).

4 Dao-Thi, M. H. *et al.* Molecular basis of gyrase poisoning by the addiction toxin CcdB. *J Mol Biol* **348**, 1091-1102, doi:10.1016/j.jmb.2005.03.049 (2005).

5 De Jonge, N. *et al.* Rejuvenation of CcdB-Poisoned Gyrase by an Intrinsically Disordered Protein Domain. *Mol Cell* **35**, 154-163, doi:10.1016/j.molcel.2009.05.025 (2009).

6 Svergun, D., Barberato, C. & Koch, M. H. J. CRYSOL - A program to evaluate x-ray solution scattering of biological macromolecules from atomic coordinates. *J Appl Crystallogr* **28**, 768-773, doi:DOI 10.1107/S0021889895007047 (1995).

7 Konarev, P. V., Volkov, V. V., Sokolova, A. V., Koch, M. H. J. & Svergun, D. I. PRIMUS: a Windows PC-based system for small-angle scattering data analysis. *J Appl Crystallogr* **36**, 1277-1282, doi:10.1107/S0021889803012779 (2003).

8 Tria, G., Mertens, H. D. T., Kachala, M. & Svergun, D. I. Advanced ensemble modelling of flexible macromolecules using X-ray solution scattering. *Iucrj* **2**, 207-217, doi:10.1107/S205225251500202x (2015).

9 Fiser, A., Do, R. K. G. & Sali, A. Modeling of loops in protein structures. *Protein Sci* **9**, 1753-1773 (2000).

10 Fiser, A. & Sali, A. ModLoop: automated modeling of loops in protein structures. *Bioinformatics* **19**, 2500-2501, doi:10.1093/bioinformatics/btg362 (2003).

11 Krivov, G. G., Shapovalov, M. V. & Dunbrack, R. L. Improved prediction of protein side-chain conformations with SCWRL4. *Proteins* **77**, 778-795, doi:10.1002/prot.22488 (2009).

12 MacKerell, A. D. & Banavali, N. K. All-atom empirical force field for nucleic acids: II. Application to molecular dynamics simulations of DNA and RNA in solution. *J Comput Chem* **21**, 105-120, doi:DOI 10.1002/(Sici)1096-987x(20000130)21:2<105::Aid-Jcc3>3.0.Co;2-P (2000).

13 MacKerell, A. D. *et al.* All-atom empirical potential for molecular modeling and dynamics studies of proteins. *J Phys Chem B* **102**, 3586-3616 (1998).

14 Mackerell, A. D., Feig, M. & Brooks, C. L. Extending the treatment of backbone energetics in protein force fields: Limitations of gas-phase quantum mechanics in reproducing protein conformational distributions in molecular dynamics simulations. *J Comput Chem* **25**, 1400-1415, doi:10.1002/jcc.20065 (2004).

15 Foloppe, N. & MacKerell, A. D. All-atom empirical force field for nucleic acids: I. Parameter optimization based on small molecule and condensed phase macromolecular target data. *J Comput Chem* **21**, 86-104, doi:10.1002/(Sici)1096-987x(20000130)21:2<86::Aid-Jcc2>3.0.Co;2-G (2000).

16 Abraham, M. J. *et al.* GROMACS: High performance molecular simulations through multi-level parallelism from laptops to supercomputers. *SoftwareX* **1,2**, 19-25, doi:http://dx.doi.org/10.1016/j.softx.2015.06.001 (2015).

17 Essmann, U. *et al.* A Smooth Particle Mesh Ewald Method. *J Chem Phys* **103**, 8577-8593, doi:DOI 10.1063/1.470117 (1995).

18 Bussi, G., Donadio, D. & Parrinello, M. Canonical sampling through velocity rescaling. *J Chem Phys* **126**, 014101, doi:10.1063/1.2408420 (2007).

19 Hess, B., Bekker, H., Berendsen, H. J. C. & Fraaije, J. G. E. M. LINCS: A linear constraint solver for molecular simulations. *J Comput Chem* **18**, 1463-1472, doi:DOI 10.1002/(Sici)1096-987x(199709)18:12<1463::Aid-Jcc4>3.0.Co;2-H (1997).
